# Establishment of a Multiplex RT-PCR Method for the Detection of Five Known Genotypes of Porcine Astroviruses

**DOI:** 10.3389/fvets.2021.684279

**Published:** 2021-06-15

**Authors:** Xin Liu, Wenchao Zhang, Dongjing Wang, Xinyue Zhu, Ying Chen, Kang Ouyang, Zuzhang Wei, Huan Liu, Weijian Huang

**Affiliations:** ^1^College of Animal Science and Technology, Guangxi University, Nanning, China; ^2^Institute of Animal Husbandry and Veterinary Medicine, Tibet Academy of Agriculture and Animal Husbandry Science, Lhasa, China; ^3^Department of Scientific Research, The First Affiliated Hospital of Guangxi University of Chinese Medicine, Nanning, China

**Keywords:** porcine astrovirus, multiplex RT-PCR, co-infection, genotype differentiation, epidemiology, Guangxi, China

## Abstract

Porcine astroviruses (PAstVs) are prevalent in pigs worldwide, and five genotypes have been reported to circulate in China. However, little is known about the coinfection status of PAstVs. For differential and simultaneous diagnoses of these five genotypes of PAstVs, a multiplex RT-PCR method was established on the basis of the *ORF2* gene of type 1 PAstV, and the *ORF1ab* genes of type two to five PAstVs. This quintuple PCR system was developed through optimization of multiplex PCR and detection sensitivity and specificity. The results showed that this multiplex RT-PCR method could specifically detect all the five PAstV genotypes without cross-reaction to any other major viruses circulating in Chinese pig farms. The detection limit of this method was as low as 10 pg of standard plasmids of each PAstV genotype. In addition, a total of 275 fecal samples collected from different districts of Guangxi, China, between April 2019 and November 2020, were tested by this newly established multiplex RT-PCR. Moreover, the sensitivity and specificity of monoplex and multiplex RT-PCR methods were compared by detecting the same set of clinical positive samples. The results revealed that PAstV1 (31/275), PAstV2 (49/275), PAstV3 (36/275), PAstV4 (41/275), and PAstV5 (22/275) were all detected, and dual (PAstV1+PAstV2, PAstV1+PAstV3, PAstV2+PAstV3, PAstV2+PAstV4, PAstV3+PAstV4, and PAstV4+PAstV5) or triple genotypes (PAstV1+PAstV2+PAstV3 and PAstV2+PAstV3+PAstV4) of coinfections were also unveiled in this study. The detection result of multiplex PCR was consistent with that of monoplex PCR. Compared with monoplex PCR, this multiplex PCR method showed obvious advantages such as time and cost efficiency and high sensitivity and specificity. This multiplex RT-PCR method offered a valuable tool for the rapid and accurate detection of PAstV genotypes circulating in pig herds and will facilitate the surveillance of PAstV coinfection status.

## Introduction

Astroviruses are non-enveloped, positive-sense, single-stranded RNA (+ssRNA) viruses whose genomes are 6–7 kb in length and contain three open reading frames (ORFs), namely, ORF1a, ORF1b, and ORF2 (1). Astrovirus could infect a wide range of hosts from birds to mammals including humans, causing diseases from asymptomatic to systematic such as diarrhea, vomiting, and virus-associated hepatitis in birds or encephalitis in human and mammals (2, 3). In 1980, porcine astrovirus (PAstV) was firstly discovered from pig feces by electron microscopy (4). Since then, PAstV was generally considered as a diarrhea-associated agent and circulated in many countries worldwide (5–7). However, polioencephalomyelitis cases have emerged in pig herds in recent years, indicating the neuro-pathogenicity and neuro-invasiveness of PAstVs (8–10). Based on the full-length ORF2 sequences, PAstV could be divided into five distinct genotypes (PAstV1–PAstV5), suggesting different genetic evolutionary ancestors of PAstV (11). The overall prevalence rates and the dominant genotypes of PAstV in different countries or districts varied on geographic locations. Xiao et al. (7) reported that 64% of fecal samples collected from US farms were detected to be positive for PAstV, and 97.2% of PAstV-positive pigs were shown to be infected by PAstV4; 80% of healthy finisher pigs from a Canadian province were found harboring PAstV at slaughter (12). In addition, 70.4% of pigs were detected to be PAstV4 positive in five European countries (13). Till now, all the five known PAstV genotypes have been detected in China (6, 14), and the overall prevalence rate ranged from 17.5% in Sichuan Province to 56.4% in Guangxi Province (6, 15, 16). Meanwhile, the prevalence rates in Thailand (6.5%) (17) and India (17.6%) (5) were lower than in other countries. Moreover, coinfections of two more PAstV genotypes or PAstV with other pig viruses were also observed (7, 14, 15). PAstV2 and PAstV5 were found in the brains of newborn piglets suffering congenital tremors (18). PAstV2 and PAstV4 were detected from the blood and fecal samples, causing viremia and circulate in pig herds (19). It is worth noting that genetic recombination events among PAstVs or other astrovirus species were frequently reported, which may contribute to the genetic diversity and evolution of PAstVs (19–24). Multiple genotypes of PAstV coinfections will further accelerate the genetic variation of this virus and bring challenges to the monitoring of PAstVs. In addition, the interspecies barrier of PAstV may not be strict. Results of genetic evolution analysis suggest that PAstV may have crossed the interspecies barrier between humans and other animals (23, 25, 26).

Considering the error-prone RNA polymerase, multi-genotype coinfections, frequent recombination events, and the zoonotic potential of PAstV, a comprehensive PAstV diagnosis method is in urgent need. It is necessary to establish an efficient and fast detection method to clarify the infection and genetic variation status of PAstV in pigs. However, the detection methods used currently are usually time-consuming and expensive. In this study, a multiplex PCR detection method was established and showed good specificity and high sensitivity. Additionally, this assay was employed to analyze a total of 275 swine fecal samples collected from different districts of Guangxi. These results provided us with a detailed PAstV infection status of swine herds in Guangxi and will facilitate the virus evolution monitoring and the development of accurate prevention strategies for PAstV.

## Materials and Methods

### Porcine Astrovirus and Major Swine Viruses

All the five genotypes of PAstV-positive samples were collected and identified by our laboratory previously and preserved at −80°C (6). The complete or partial genomic sequences of these positive samples are available in GenBank under following accession numbers: NC_025379 for PAstV1, KY412124 for PAstV2, KY412129 for PAstV3, KY412125 for PAstV4, and MH064173 for PAstV5. Porcine enterovirus G (EV-G), porcine Seneca virus [Seneca Valley virus (SVV)] (27), porcine pseudorabies virus (PRV), classical swine fever virus (CSFV), porcine reproductive and respiratory syndrome virus (PRRSV) (28), porcine epidemic diarrhea virus (PEDV) (29), porcine transmissible gastroenteritis virus (TGEV), and porcine rotavirus (PoRV) were all isolated and identified by our laboratory and stored at −80°C. The total RNA was extracted using TRIzol reagent (Takara, Dalian, China) and subjected to reverse transcription for first cDNA synthesis with the PrimeScript RT reagent (Takara, Dalian, China) following the manufacturer's instructions. The genomic DNA of PRV was extracted by TIANamp Virus DNA/RNA Kit (TIANGEN, Beijing, China) according to the Kit instructions. The obtained cDNAs and viral genomic DNA were stored at −80°C until use.

### Experimental Design

In this study, we designed a multiplex RT-PCR method to identify the five known genotypes of PAstV in a single reaction tube. In short, the total RNA of fecal samples was extracted, and then the cDNA was synthesized by reverse transcription using hexamer random primers. The cDNAs and the primers specific for PAstV1, PAstV2, PAstV3, and PAstV4 and PAstV5 were added into the PCR mixture. PCR products were observed under UV light after 1.5% agarose gel electrophoresis. The genotypes were identified according to the length of PCR fragments.

### Primer Design and Standard Preparation

Based on the highly conserved regions of PAstV representative strains in GenBank, genotype-specific primer sets targeting the *OFR2* gene of PAstV1, and the *ORF1ab* genes of PAstV2, PAstV3, PAstV4, and PAstV5 were designed by Oligo 6.0 along with National Center for Biotechnology Information (NCBI) primer-BLAST comparison. All these primers were synthesized by Shanghai Sangon Biotech Co., Ltd. (Shanghai, China) and diluted with distilled deionized water (ddH_2_O) to a concentration of 10 μmol/L and stored at −20°C for later utilization. The primer sequences and the respective amplification lengths are shown in Table 1.

**Table 1:** Multiplex primers used in this study.

**Genotype**	**Primer name**	**Sequence (5^**′**^-3^**′**^)**	**GenBank accession**	**Product size (bp)**	**Target gene**	**Position**
PAstV1	PAstV1-F	GGCCGTGGCAGGAGCAGATC	NC_025379	124	*ORF2*	4,300–4,424
	PAstV1-R	GACTGAGGTTTACCCCGTCT				
PAstV2	PAstV2-F	ACCACCGCGCAGGAGG	NC_023674	573	*ORF1ab*	2,537–3,109
	PAstV2-R	TGTTGYTCAAGRGCAGC				
PAstV3	PAstV3-F	GATGTGATGACCCTCTATGGG	NC_019494	175	*ORF1ab*	3,879–4,053
	PAstV3-R	GCCGGTCAAGCATCTCATCAG				
PAstV4	PAstV4-F	TGGGGTCCTGAAGCATTTGC	JF713713	485	*ORF1ab*	2,777–3,261
	PAstV4-R	AATGGGGACCATCCACA				
PAstV5	PAstV5-F	AATGTGCGKGTGAAAGA	JX556693	305	*ORF1ab*	3,319–3,623
	PAstV5-R	TGAAATGTGACTTCACCTGA				

In order to build detection standards of the multiplex PCR assay, the synthesized cDNAs obtained from PAstV-positive samples were used as templates and mixed with the primer sets for the individual genotypes to amplify all the fragments of the five genotypes. A 50 μl PCR system was built as follows: 25 μl 2 × Premix Taq (Takara, Dalian, China), 2.5 μl cDNA template (about 100 ng/μl), primer sets at a final concentration of 1.0 μmol/L, and ddH_2_O were added to a final volume of 50 μl. The PCRs were conducted according to the manufacturer's instructions. PCR products were stained with ethidium bromide (EB), separated by 1.5% agarose gel electrophoresis, and visualized under UV light. The PCR products were further gel purified and cloned into pMD18-T vector (Takara, Dalian, China) according to manufacturer's instructions. These constructed plasmids were transformed into competent *Escherichia coli* DH5α for propagation. The recombinant plasmid DNAs were extracted and purified by TIANprep Mini Plasmid Kit (TIANGEN, Beijing, China) according to kit instructions and sequenced with M13 primers. The plasmid DNAs were quantified spectrophotometrically by NanoDrop 2000 (Thermo Fisher Scientific, Waltham, MA, USA) and diluted to 100 ng/μl. Subsequently, the standards were 10-fold diluted in ddH_2_O, resulting a concentration gradient of 10 ng/μl, 1 ng/μl, 100 pg/μl, 10 pg/μl, 1 pg/μl, and 0.1 pg/μl and used as templates to evaluate the analytic sensitivity of the monoplex and multiplex RT-PCR assays.

### Establishment of Multiplex PCR

For the multiplex RT-PCR assay development, a duplex PCR was firstly established with the primer sets for type 1 and type 2 PAstVs, using the corresponding standards as templates (100 ng each). A 20 μl PCR system was built as follows: 10 μl 2 × Premix Taq, 1 μl standards (~100 ng), primer sets at a final concentration of 1.0 μmol/L, and ddH_2_O were added to a final volume of 20 μl. The PCRs were conducted under the following conditions: 30 cycles of 10 s at 98°C; 30 s at 55°C, 1 min at 72°C, and final extension of 45 s at 72°C The primer sets of type three to five PAstV and its standards were added to the former established duplex (PAstV1–2), triplex (PAstV1–3), and quadruple (PAstV1–4) PCR assays one by one to establish the final quintuple PCR to detect all the genotypes. For a better output of the multiplex PCR assay, the reactions conditions were optimized by varying a single parameter, while other parameters were fixed as described by Ding et al. (30). The primer concentration for each target ranged from 0.2 to 1.0 μmol/L The annealing temperatures (53–57°C) were also tested. In this way, the primer concentration and annealing temperature were optimized. All PCR amplifications were carried out under the optimized conditions in one tube, and the PCR products were visualized on 1.5% agarose gel.

### The Sensitivity of the Multiplex RT-PCR

The sensitivity of multiplex RT-PCR detection was evaluated by detecting 10-fold (10 ng, 1 ng, 100 pg, 10 pg, 1 pg, and 0.1 pg) diluted standard plasmids of PAstV1, PAstV2, PAstV3, PAstV4, and PAstV5, respectively. The same amounts of standards of each genotype were combined and used as templates for PCR with the optimized reaction system. In addition, the sensitivity of the monoplex RT-PCR was also tested. The standards of each genotype were added to a separate RT-PCR tube as an amplification template.

### The Specificity Test of Multiplex PCR

The established RT-PCR system was used to amplify the cDNAs or DNA templates of EV-G, SVV, PRRSV, PEDV, TGEV, PoRV, CSFV, and PRV-positive samples. Primer sets targeting these viruses were used as internal control. The primer sequences and the respective amplification lengths are shown in Table 2. The specificity of the method was verified using the mixed standards (100 ng each) as a positive control.

**Table 2:** Detection primers used for specificity analysis.

**Viruses**	**Sequence (5^**′**^-3^**′**^)**	**Target genes**	**Product size (bp)**	**Reference**
PRV	F: CGGCTTCCACTCGCAGCTCTTCTC	*gE*	388	MN443981.1
	R: TCTGGGTCATCACGAGCACGTACAGC			
CSFV	F: ACAGCCACGATTTGCAACTGTATG	*E2*	347	FJ598612.1
	R: TCTCAGAGTTGTTGGGCTCACTGC			
PoRV	F: GATGCTAG GACAAAATTG	*VP6*	309	MG066585.1
	R: CGCTTCAGATTGCGGAGCTAC			
TGEV	F: GACAAACTCGCTATCGCATGGTG	*N*	638	KU981074.1
	R: CACAGATGGAACACATTCAGCCAG			
PEDV	F: ATTCGCTGGCGCATGCGCCGTGGTG	*N*	509	JN601062.1
	R: ACAGCAGCCACCAGATCATCGCGTG			
EV-G	F: AGACTGGAGCTAGCTCCACTGCTAG	*VP1*	302	MT274669.1
	R: GACCTGGACTTGAACTGGGTGCTGT			
SVV	F: CACCTGACTGCCCACAGAGTCCCTGT	*VP1*	813	MK039162.1
	R: CCGCCACGTGCTTTACAGCGGTGCTT			
PRRSV	F: TGTATCGTGCCGTTCTATCTTGCTGT	*ORF5*	547	EF635006.1
	R: AGAGACGACCCCATTGTTCCGCTG			

### Detection of Clinical Samples

A total of 275 fecal samples were collected from Nanning, Chongzuo, Liuzhou, and Guigang in Guangxi Province between April 2019 and November 2020. All these samples (about 100 mg each) were mixed with 500 μl of sterile phosphate-buffered saline (PBS) and centrifuged at 2,000 × g for 20 min at 4°C. About 300 μl of the supernatants was collected and subjected to RNA extraction by TRIzol reagent and following cDNA synthesis as manufacturer's instructions. The cDNAs (about 100 ng/μl) were then subjected to PCR amplification by the established multiplex and monoplex in a 20 μl reaction mixture: 10 μl 2 × Premix Taq, 3 μl cDNA, primer sets at a final concentration of 0.8 μmol/L, and ddH_2_O were added to a final volume of 20 μl. The detection results were compared to evaluate the detection consistency between monoplex and multiplex PCR methods established in this study. The standard plasmids were used as a positive control and determination criteria of the multiplex PCR results.

## Results

### Establishment of Multiplex RT-PCR Method

**Figure 1:**
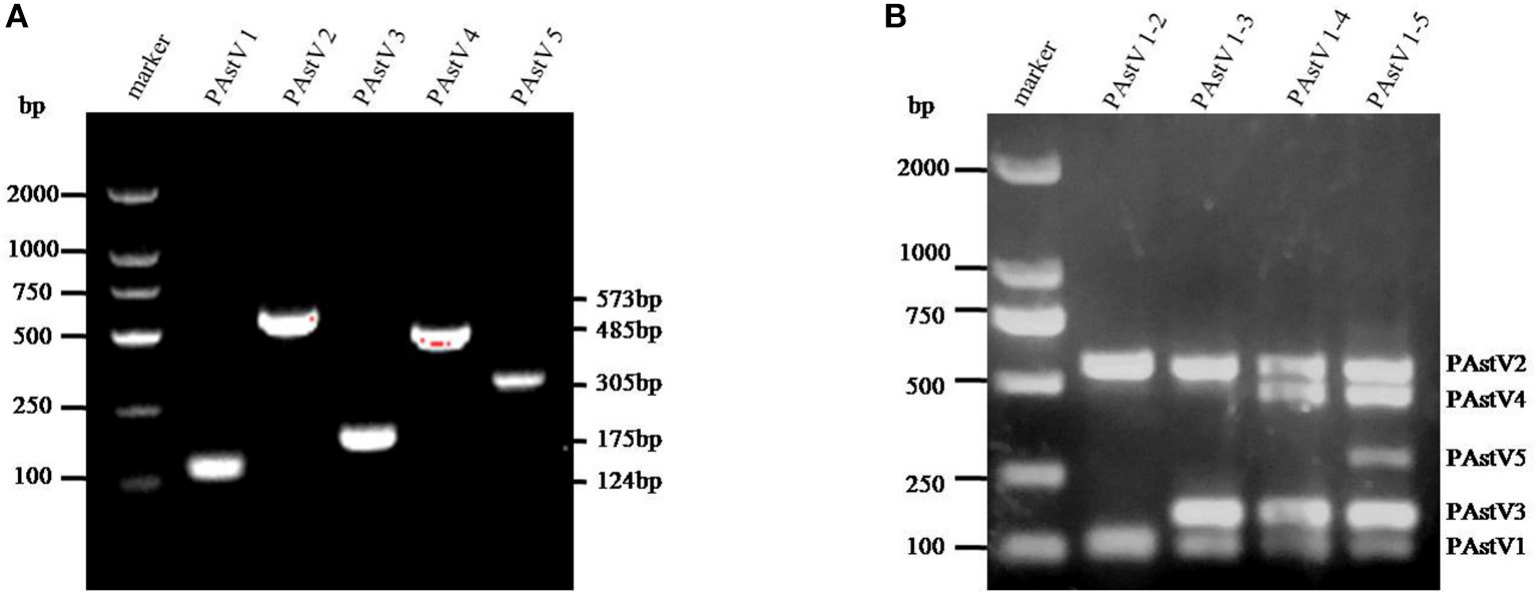
The multiplex RT-PCR assay is well-established. **(A)** Total RNA was extracted from the clinical positive samples with TRIzol reagent and subjected to reverse transcription with hexamer random primer. The cDNAs were amplified with primers targeting genes of each genotype described in Table 1. **(B)** Multiplex RT-PCR was developed for the detection of all these five known porcine astrovirus (PAstV) genotypes. The prepared standard plasmids and corresponding primer sets were added one by one, constituting duplex, triplex, quadruple, and quintuple PCR mixtures.

The monoplex RT-PCR result showed that the fragments at expected sizes of each genotype (124 bp for PAstV1, 573 bp for PAstV2, 175 bp for PAstV3, 485 bp for PAstV4, and 305 bp for PAstV5) were successfully amplified from the stored positive samples (Figure 1A). In addition, neither non-specific bands nor primer dimers appeared on the agarose gel, indicating high amplification quality and specificity of these primer sets (Figure 1A). Next, the standards and primer sets of type one to five PAstVs were added to the reaction tube one by one, and the results demonstrated that all these target genes were well-amplified without any interference, indicating good amplification and high efficacy of this multiplex RT-PCR method (Figure 1B).

### Optimization of the Multiplex RT-PCR Conditions

**Figure 2:**
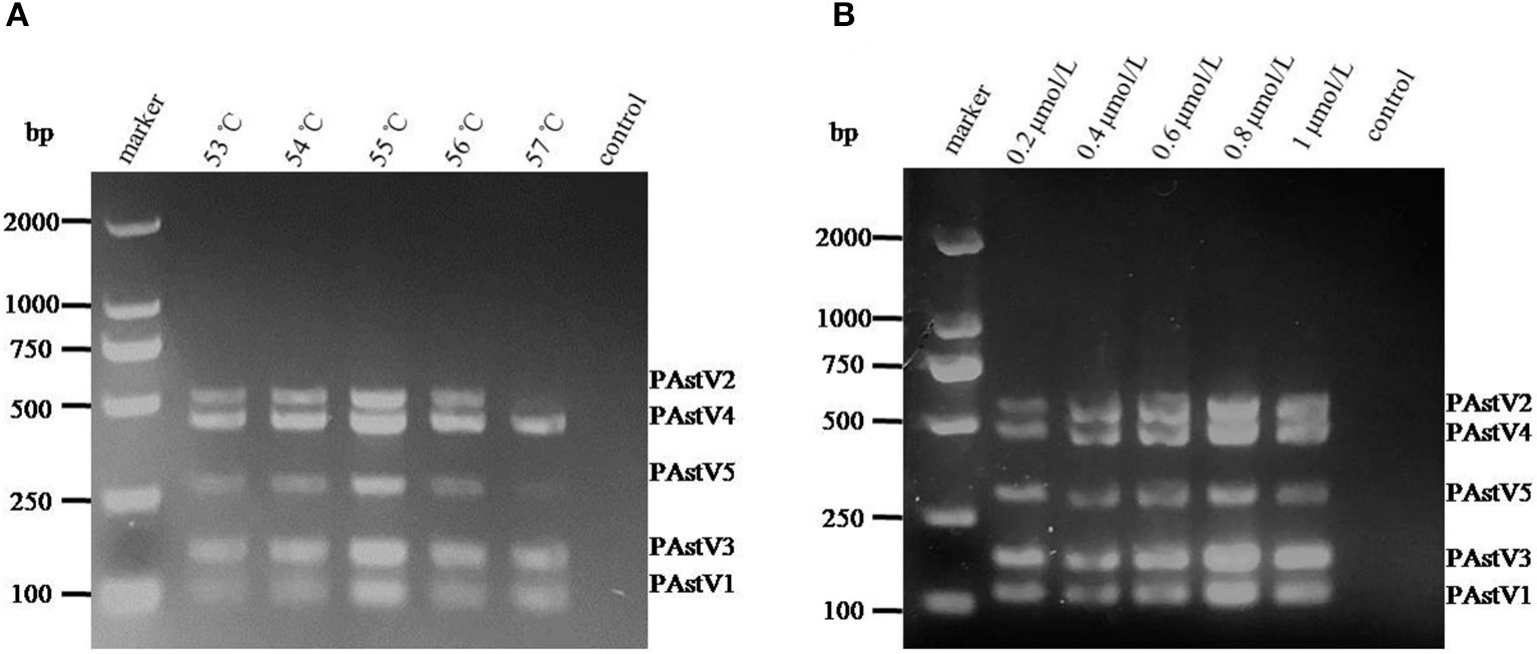
Optimization of the multiplex RT-PCR conditions. **(A)** The standard plasmids of each porcine astrovirus (PAstV) genotype (10 ng each) were mixed and used as template for PCR amplification with combined primer sets at a final concentration of 1 μmol/L. **(B)** The standard plasmids (10 ng each) were combined with the primer sets at different concentrations (0.2–1.0 μmol/L) and amplified at an annealing temperature of 55°C.

With the use of the standard plasmids (10 ng each) as templates, the PCR annealing temperatures and primer concentrations were optimized in this study. On equal conditions, annealing temperature at 55°C could obtain the best detection result (Figure 2A). Meanwhile, the optimal primer concentration was revealed to be 0.8 μmol/L (Figure 2B).

### The Sensitivity of the Multiplex RT-PCR

**Figure 3:**
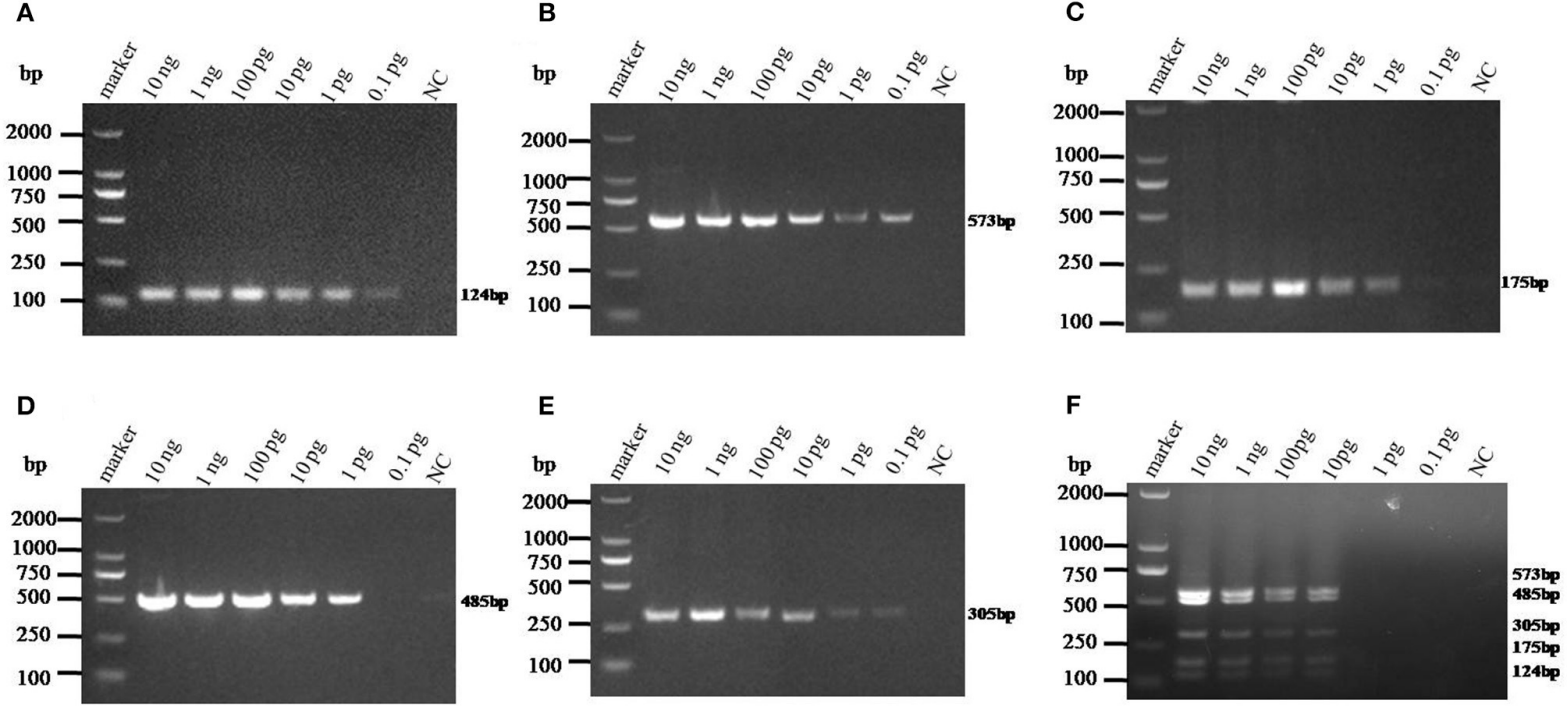
Sensitivity analysis of the monoplex and multiplex PCR method established in this study. Equal amounts of standards of each genotype (10 ng−0.1 pg) were added to the single or multiplex PCR mixture and amplified at the optimized thermo cycle condition. **(A–E)** Monoplex PCR sensitivity analysis of PAstV1, PAstV2, PAstV3, PAstV4, and PAstV5, respectively. **(F)** Multiplex PCR sensitivity analysis.

The sensitivity of monoplex RT-PCR to each genotype was firstly investigated. The results showed that the standards of PAstV1, PAstV2, and PAstV5 were detectable with a minimum amount of 0.1-pg standards, while the standards of PAstV3 and PAstV4 could be detected as low as 1 pg (Figures 3A–E), indicating high sensitivity of the designed primer sets to each genotype. When the sensitivity of multiplex RT-PCR is measured, all the primers are mixed at the optimal concentration to prepare a PCR mixture, which was used to detect pooled standards of each genotype at the indicated amounts (10 ng−0.1 pg). The results showed that the detection limit of this method was as low as 10-pg standards of all the five genotypes of PAstVs (Figure 3F), indicating high sensitivity of the multiplex RT-PCR for PAstV detection.

### The Specificity of the Multiplex RT-PCR

**Figure 4:**
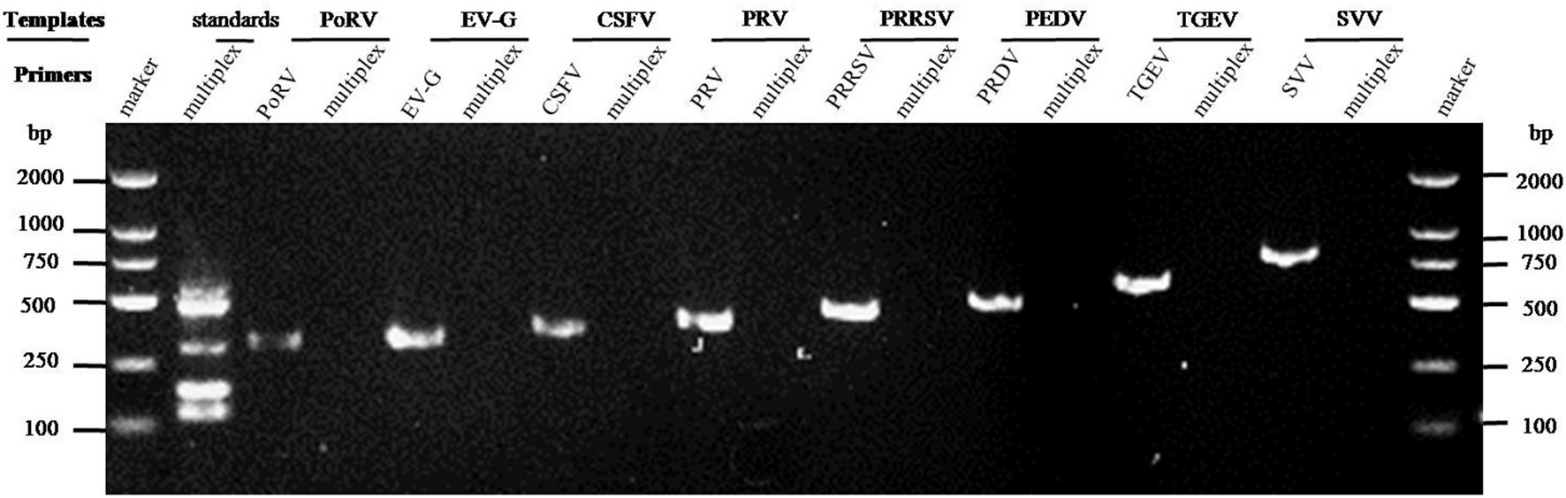
Specificity analysis of the multiplex RT-PCR method established in this study. The cDNA or DNA obtained from common swine viruses circulating in Chinese pig herds was used as templates to validate the specificity of the multiplex RT-PCR assay. The porcine astrovirus (PAstV) standards were mixed and used as positive control. Primers specific to other individual viruses were employed as internal controls. Primer sequences are shown in Table 2.

The cDNAs of EV-G, PRRSV, SVV, CSFV, PEDV, PoRV, TGEV, and the DNA template of the PRV samples were used to detect specificity by the established RT-PCR method. The results showed that five target fragments were obtained when standard plasmids were used as template. Meanwhile, no bands were detected if templates were replaced by other common viruses' cDNA or DNA (Figure 4). This method did not cross-react with other major swine pathogens, indicating good specificity of this multiplex PCR method.

### Detection of Field Samples Using the Multiplex RT-PCR

**Figure 5:**
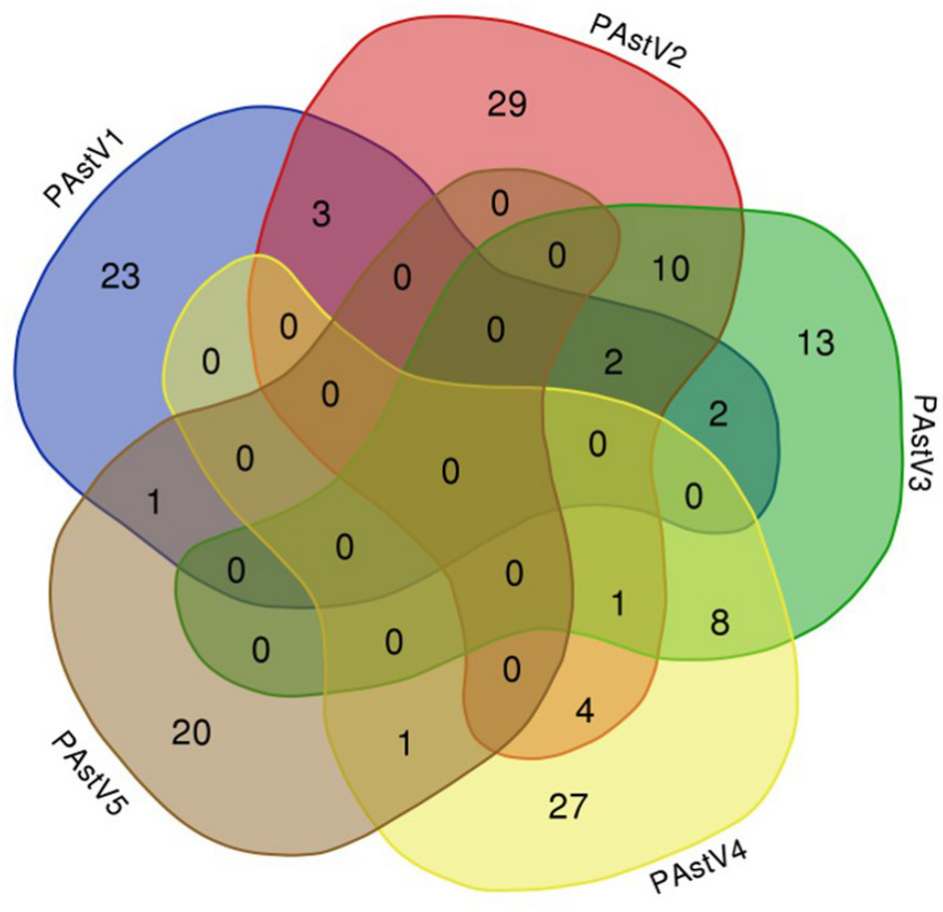
Venn diagram showing the total and proportion of positive samples for each porcine astrovirus (PAstV) genotype. The overlapping areas indicate the total samples that were positive in one to three different genotypes by multiplex RT-PCR.

**Figure 6:**
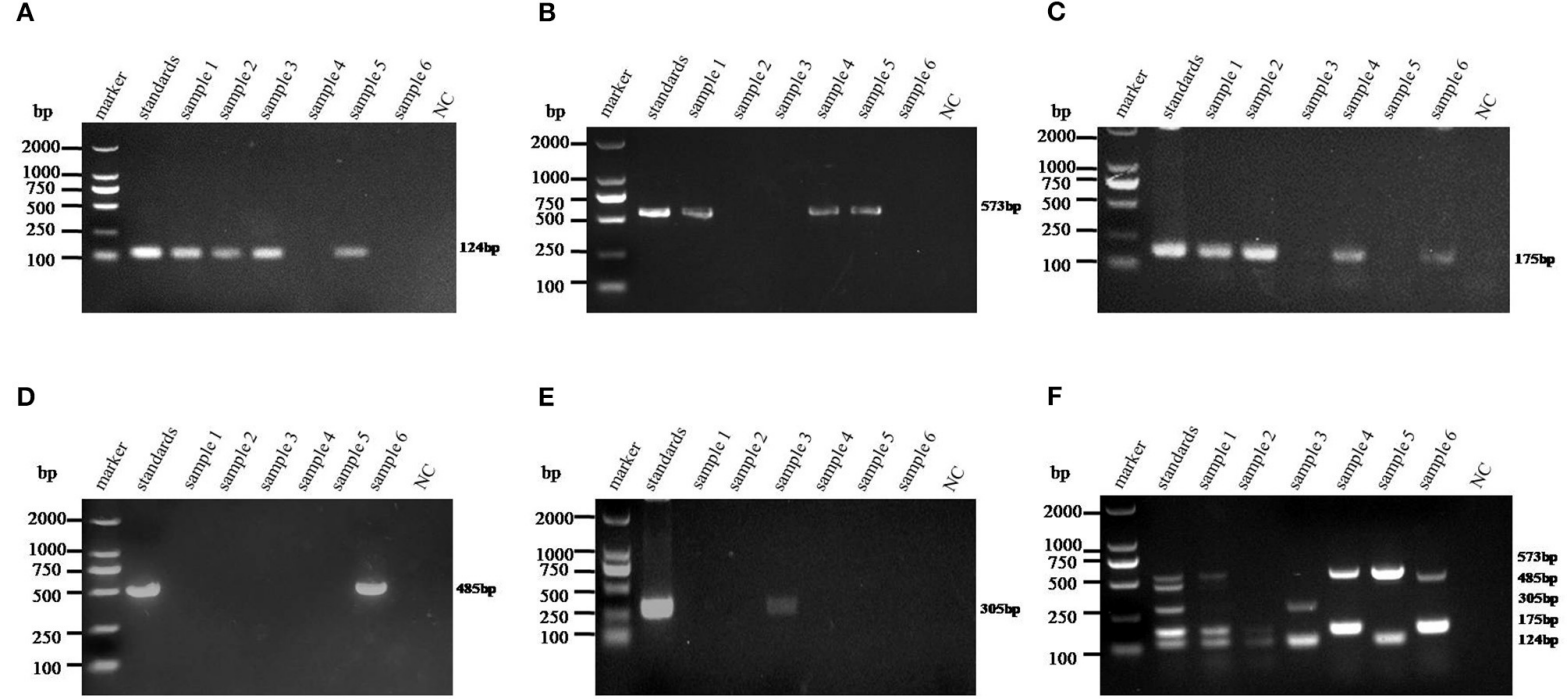
Detection consistency analysis of multiplex and monoplex PCR used in this study. The standards of each genotype were mixed as a template pool and used as a positive control. The cDNAs of selected samples were used as templates for monoplex and multiplex PCR in the optimized PCR system. **(A–E)** Porcine astrovirus (PAstV) one to five genotype monoplex PCR results of the selected positive samples. **(F)** Multiplex PCR result of the selected positive samples. The indicated sizes of each genotype are shown in the right panel.

The 275 fecal samples from different districts of Guangxi Province were detected by this newly established multiplex RT-PCR method. The results showed that the overall positive rate of PAstV infection was 46.9% (129/275); and PAstV1 (31/275), PAstV2 (49/275), PAstV3 (36/275), PAstV4 (41/275), and PAstV5 (22/275) were all found circulating in pig herds in Guangxi Province (Figure 5). In addition, dual-genotype infections such as PAstV1+PAstV2 (3/275), PAstV2+PAstV3 (10/275), PAstV3+PAstV4 (8/275), and PAstV4+PAstV2 (4/275) and even triple genotype of PAstV infections, such as PAstV1+PAstV2+PAstV3 (2/275) and PAstV2+PAstV3+PAstV4 (1/275), were also detected (Figure 5). Moreover, as shown in Table 3, the infection rate of sucking piglets (77.8%) is much higher than that of other age groups, indicating that the younger groups are more susceptible to PAstVs infection. Meanwhile, the overall infection rates of PAstV2 (17.8%, 49/275) and PAstV4 (14.9%, 41/275) were moderately higher than those of other types, indicating the dominance of these genotypes in Guangxi. cDNAs of the same set of positive samples were used as temples for detection consistency analysis. As shown in Figure 6, the detection result of multiplex PCR is in concordance with that of monoplex PCR, indicating good reliability of this method.

**Table 3:** Results of field samples detected by the multiplex RT-PCR.

**Age groups**	**Sample number**	**Positive rate (positive number)**	**Number of positive samples**				
			**PAstV1**	**PAstV2**	**PAstV3**	**PAstV4**	**PAstV5**
Suckling pig	45	77.8% (35)	5	13	16	16	1
Nursery pigs	45	48.8% (22)	10	12	15	10	1
Growing and fattening pigs	35	57.1% (20)	3	10	5	7	0
Lactating sow	80	53.7% (43)	5	14	0	7	20
Pregnant sow	40	10% (4)	3	0	0	1	0
Backup pigs	30	16.6% (5)	5	0	0	0	0
Total	275	46.9% (129)	31	49	36	41	22

## Discussion

PAstV has been circulating in many countries around the world. In the recent decade, with the aid of improved sequencing techniques such as high-throughput sequencing, increasing novel clades of astroviruses have been discovered (31). For quite some time, PAstV was considered as a low-pathogenic virus causing a short-term mild diarrhea (32, 33). However, emerging cases of PAstV associated enteritis or polioencephalomyelitis were reported and attracted public attention in recent years (8, 9, 34, 35). As an enteric virus, PAstVs were more frequently detected in the pig herds, and the prevalent rates of PAstV were usually reported much higher than those of other diarrheal viruses such as PEDV, TGEV, and porcine deltacoronavirus (PDCoV) (30, 36). Besides, the high genetic variability and possible recombination events of PAstVs further remind people to develop comprehensive methods for astrovirus diagnosis and epidemiological investigation (23, 37).

At present, methods used for PAstV diagnosis mostly stay at the level of single RT-PCR or quantitative RT-PCR detection (6, 38). Although more advanced detection methods such as nanofluidic PCR, microarrays, or high-throughput sequencing are valuable assets for the diagnosis of astrovirus (39), there are some disadvantages such as being expensive and time-consuming and requiring instruments and experimenters, limiting their popularization and field application. Multiplex PCR/RT-PCR is still widely used in veterinary diagnostic centers at present. This was mainly owing to its cost-efficiency, simple procedures, and time-efficiency. In addition, multiplex PCR technology can detect multiple pathogens at the same time, which is of great value in differential diagnosis, especially in veterinary medicine.

As mentioned previously, coinfections of multiple genotypes of PAstVs in a pig farm, even in an individual pig, were reported (7, 16). Moreover, five known genotypes of PAstVs were detected in China (6, 14, 40). However, the methods used in those studies could not differentiate the genotypes at the first time. Based on genotype-specific primer sets, multiplex RT-PCR method was built in this study and showed good performance in PAstV genotype differentiation. The detection limit of the multiplex PCR method established in this experiment for PAstV1, PAstV2, PAstV3, PAstV4, and PAstV5 is 10 pg of standard plasmids, indicating high sensitivity and good field applicability. Of the 275 collected fecal samples, all five known genotypes of PAstV were detected, and dual or triple genotypes of PAstV coinfections were also unveiled in this study (Figure 5). Meanwhile, PAstV2 and PAstV4 were shown to be the dominant genotypes in Guangxi, which is consistent to our lab's previously results (6). As for humans, infant and young children were major victims of astrovirus infection (41, 42). In this study, the infection rates of pigs at lactation and nursery stage were also higher than those of grown pigs (Table 2). Pathogenic studies have shown that PAstV infection could cause diarrhea and growth retardation, which could lead to economic losses and cannot be ignored in large-scale pig industries (32, 33). Moreover, coinfections of PAstV with other swine viruses were also reported, which pointed out that PAstVs were more intended to co-infect with other viruses, and immunosuppressive viruses such as CSFV could benefit from the replication of PAstV (13, 14, 43). All this reminds us that PAstV infections could be a landmine if it is not well-controlled. However, comprehensive understanding of all types of PAstV epidemiology, which is of great value for infectious disease control, is not available. The multiplex RT-PCR method developed in this study specifically targets all types of PAstVs and performed well in detection specificity and sensitivity, providing a valuable tool for PAstV clinical diagnosis and understanding the full picture of PAstV infections.

## Data Availability Statement

The datasets presented in this study can be found in online repositories. The names of the repository/repositories and accession number(s) can be found in the article/supplementary material.

## Ethics Statement

The animal study was reviewed and approved by Ethics Committee of Animal Experiments of Guangxi University (protocol number: GXU2018-044).

## Author Contributions

HL and WH designed the experiments. XL and WZ are major contributors for experimental implementation. XZ is mainly responsible for sample collection and helped perform some experiments. HL and XL wrote the manuscript. All authors have read and approved the final manuscript.

## Conflict of Interest

The authors declare that the research was conducted in the absence of any commercial or financial relationships that could be construed as a potential conflict of interest.

## References

[1] Mendez E Arias CF. Astroviruses. In: Fields Virology. 6th ed. New York, NY: Lippincott Williams &Wilkins (2013). p. 609–28.

[2] Cortez V Meliopoulos VA Karlsson EA Hargest V Johnson C Schultz-Cherry S. Astrovirus biology and pathogenesis. Annu Rev Virol. (2017) 4:327–48. 10.1146/annurev-virology-101416-041742 28715976

[3] Johnson C Hargest V Cortez V Meliopoulos VA Schultz-Cherry S. Astrovirus pathogenesis. Viruses. (2017) 9:22. 10.3390/v9010022 PMC5294991 28117758

[4] Bridger JC. Detection by electron microscopy of caliciviruses, astroviruses and rotavirus-like particles in the faeces of piglets with diarrhoea. Vet Rec. (1980) 107:532–3. 6258286

[5] Kattoor JJ Malik YS Saurabh S Sircar S Vinodhkumar OR Bora DP . First report and genetic characterization of porcine astroviruses of lineage 4 and 2 in diarrhoeic pigs in India. Transbound Emerg Dis. (2019) 66:47–53. 10.1111/tbed.13058 30379411

[6] Qin Y Fang Q Li X Li F Liu H Wei Z. Molecular epidemiology and viremia of porcine astrovirus in pigs from Guangxi province of China. BMC Vet Res. (2019) 15:471. 10.1186/s12917-019-2217-x 31881886 PMC6935060

[7] Xiao CT Giménez-Lirola LG Gerber PF Jiang YH Halbur PG Opriessnig T. Identification and characterization of novel porcine astroviruses (PAstVs) with high prevalence and frequent co-infection of individual pigs with multiple PAstV types. J Gen Virol. (2013) 94:570–82. 10.1099/vir.0.048744-0 23223616

[8] Matias Ferreyra FS Bradner LK Burrough ER Cooper VL Derscheid RJ Gauger PC. Polioencephalomyelitis in domestic swine associated with porcine astrovirus type 3. Vet Pathol. (2020) 57:82–9. 10.1177/0300985819875741 31551018

[9] Arruda B Arruda P Hensch M Chen Q Zheng Y Yang C. Porcine astrovirus type 3 in central nervous system of swine with polioencephalomyelitis. Emerg Infect Dis. (2017) 23:2097–100. 10.3201/eid2312.170703 29148383 PMC5708247

[10] Boros A Albert M Pankovics P Biro H Pesavento PA Phan TG. Outbreaks of neuroinvasive astrovirus associated with encephalomyelitis, weakness, and paralysis among weaned pigs, Hungary. Emerg Infect Dis. (2017) 23:1982–93. 10.3201/eid2312.170804 29148391 PMC5708238

[11] Wu H Bao Z Mou C Chen Z Zhao J. Comprehensive analysis of codon usage on porcine astrovirus. Viruses. (2020) 12:991. 10.3390/v12090991 32899965 PMC7552017

[12] Luo Z Roi S Dastor M Gallice E Laurin MA L'Homme Y. Multiple novel and prevalent astroviruses in pigs. Vet Microbiol. (2011) 149:316–23. 10.1016/j.vetmic.2010.11.026 21159453 PMC7172684

[13] Zhou W Ullman K Chowdry V Reining M Benyeda Z Baule C. Molecular investigations on the prevalence and viral load of enteric viruses in pigs from five European countries. Vet Microbiol. (2016) 182:75–81. 10.1016/j.vetmic.2015.10.019 26711031 PMC7125590

[14] Su M Qi S Yang D Guo D Yin B Sun D. Coinfection and genetic characterization of porcine astrovirus in diarrheic piglets in china from 2015 to 2018. Front Vet Sci. (2020) 7:462. 10.3389/fvets.2020.00462 32923463 PMC7456941

[15] Cai Y Yin W Zhou Y Li B Ai L Pan M. Molecular detection of Porcine astrovirus in Sichuan Province, China. Virol J. (2016) 13:6. 10.1186/s12985-015-0462-6 26739067 PMC4704384

[16] Xiao CT Luo Z Lv SL Opriessnig T Li RC Yu XL. Identification and characterization of multiple porcine astrovirus genotypes in Hunan province, China. Arch Virol. (2017) 162:943–52. 10.1007/s00705-016-3185-0 27990567

[17] Kumthip K Khamrin P Saikruang W Kongkaew A Vachirachewin R Ushijima H. Detection and genetic characterization of porcine astroviruses in piglets with and without diarrhea in Thailand. Arch Virol. (2018) 163:1823–9. 10.1007/s00705-018-3806-x 29569070

[18] Blomström AL Ley C Jacobson M. Astrovirus as a possible cause of congenital tremor type AII in piglets? Acta veterinaria Scandinavica. (2014) 56:82. 10.1186/s13028-014-0082-y 25510194 PMC4271328

[19] Brnić D Prpić J Keros T Roić B Starešina V Jemeršić L. Porcine astrovirus viremia and high genetic variability in pigs on large holdings in Croatia. Infect Genet Evol J Mol Epidemi Evol Genet Infect Dis. (2013) 14:258–64. 10.1016/j.meegid.2012.12.027 23313832

[20] Ito M Kuroda M Masuda T Akagami M Haga K Tsuchiaka S. Whole genome analysis of porcine astroviruses detected in Japanese pigs reveals genetic diversity and possible intra-genotypic recombination. Infect Genet Evol J Mol Epidemi Evol Genet Infect Dis. (2017) 50:38–48. 10.1016/j.meegid.2017.02.008 28189887

[21] Lv SL Zhang HH Li JY Hu WQ Song YT Opriessnig T. High genetic diversity and recombination events of porcine astrovirus strains identified from ill and asymptomatic pigs in 2017, Hunan Province, China. Virus Genes. (2019) 55:673–681. 10.1007/s11262-019-01692-w 31372920

[22] Amimo JO Machuka EM Abworo EO Vlasova AN Pelle R. Whole genome sequence analysis of Porcine astroviruses reveals novel genetically diverse strains circulating in East African smallholder pig farms. Viruses. (2020) 12:1262. 10.3390/v12111262 33167568 PMC7694451

[23] Ulloa JC Gutiérrez MF. Genomic analysis of two ORF2 segments of new porcine astrovirus isolates and their close relationship with human astroviruses. Can J Microbiol. (2010) 56:569–77. 10.1139/W10-042 20651856

[24] Lan D Ji W Shan T Cui L Yang Z Yuan C. Molecular characterization of a porcine astrovirus strain in China. Arch Virol. (2011) 156:1869–75. 10.1007/s00705-011-1050-8 21688105 PMC7086730

[25] Pankovics P Boros Á Kiss T Delwart E Reuter G. Detection of a mammalian-like astrovirus in bird, European roller (*Coracias garrulus*). Infect Genet Evol J Mol Epidemi Evol Genet Infect Dis. (2015) 34:114–21. 10.1016/j.meegid.2015.06.020 26096774

[26] Mor SK Chander Y Marthaler D Patnayak DP Goyal SM. Detection and molecular characterization of *Porcine astrovirus* strains associated with swine diarrhea. J Vet Diagn Invest. (2012) 24:1064–7. 10.1177/1040638712458781 22956487

[27] Wang H Niu C Nong Z Quan D Chen Y Kang O. Emergence and phylogenetic analysis of a novel Seneca Valley virus strain in the Guangxi Province of China. Res Vet Sci. (2020) 130:207–11. 10.1016/j.rvsc.2020.03.020 32200161

[28] Wang J Lin S Quan D Wang H Huang J Wang Y. Full genomic analysis of new variants of porcine reproductive and respiratory syndrome virus revealed multiple recombination events between different lineages and sublineages. Front Vet Sci. (2020) 7:603. 10.3389/fvets.2020.00603 33134336 PMC7511543

[29] Lu Y Su X Du C Mo L Ke P Wang R. Genetic diversity of Porcine epidemic diarrhea virus with a naturally occurring truncated ORF3 gene found in Guangxi, China. Frontiers Vet. Sci. (2020) 7:435. 10.3389/fvets.2020.00435 32793651 PMC7393948

[30] Ding G Fu Y Li B Chen J Wang J Yin B. Development of a multiplex RT-PCR for the detection of major diarrhoeal viruses in pig herds in China. Transbound Emerg Dis. (2020) 67:678–85. 10.1111/tbed.13385 31597013 PMC7168528

[31] Donato C Vijaykrishna D. The broad host range and genetic diversity of mammalian and avian astroviruses. Viruses. (2017) 9:102. 10.3390/v9050102 28489047 PMC5454415

[32] Fang Q Wang C Liu H Wu Q Liang S Cen M. Pathogenic characteristics of a Porcine astrovirus strain isolated in China. Viruses. (2019) 11:1156. 10.3390/v11121156 31847270 PMC6949928

[33] Indik S Valicek L Smid B Dvorakova H Rodak L. Isolation and partial characterization of a novel porcine astrovirus. Vet Microbiol. (2006) 117:276–83. 10.1016/j.vetmic.2006.06.020 16879934

[34] Opriessnig T Xiao CT Halbur PG. Porcine astrovirus type 5-associated enteritis in pigs. J Comp Pathol. (2020) 181:38–46. 10.1016/j.jcpa.2020.09.014 33288149

[35] Rawal G Ferreyra FM Macedo NR Bradner LK Harmon KM Allison G. Ecology of Porcine astrovirus type 3 in a herd with associated neurologic disease. Viruses. (2020) 12:992. 10.3390/v12090992 32906600 PMC7552043

[36] Shi Y Li B Tao J Cheng J Liu H. The complex co-infections of multiple porcine diarrhea viruses in local area based on the luminex xTAG multiplex detection method. Front Vet Sci. (2021) 8:602866. 10.3389/fvets.2021.602866 33585617 PMC7876553

[37] Zhao C Chen C Li Y Dong S Tan K Tian Y. Genomic characterization of a novel recombinant porcine astrovirus isolated in northeastern China. Arch Virol. (2019) 164:1469–73. 10.1007/s00705-019-04162-8 30868264

[38] Goecke NB Hjulsager CK Kongsted H Boye M Rasmussen S Granberg F. No evidence of enteric viral involvement in the new neonatal porcine diarrhoea syndrome in Danish pigs. BMC Vet Res. (2017) 13:315. 10.1186/s12917-017-1239-5 29115952 PMC5678564

[39] Pérot P Lecuit M Eloit M. Astrovirus diagnostics. Viruses. (2017) 9:10. 10.3390/v9010010 PMC5294979 28085120

[40] Chu DK Poon LL Guan Y Peiris JS. Novel astroviruses in insectivorous bats. J Virol. (2008) 82:9107–14. 10.1128/JVI.00857-08 18550669 PMC2546893

[41] Olortegui MP Rouhani S Yori PP Salas MS Trigoso DR Mondal D. Astrovirus infection and diarrhea in 8 countries. Pediatrics. (2018). 141:e20171326. 10.1542/peds.2017-1326 29259078 PMC9923568

[42] Naficy AB Rao MR Holmes JL Abu-Elyazeed R Savarino SJ Wierzba TF. Astrovirus diarrhea in Egyptian children. J Infect Dis. (2000) 182:685–90. 10.1086/315763 10950760

[43] Mi S Guo S Xing C Xiao C He B Wu B. Isolation and characterization of porcine astrovirus 5 from a classical swine fever virus-infected specimen. J Virol. (2020) 95:e01513–20. 10.1128/JVI.01513-20 33115877 PMC7944439

